# Partnership for fragility bone fracture care provision and prevention program (P4Bones): study protocol for a secondary fracture prevention pragmatic controlled trial

**DOI:** 10.1186/1748-5908-8-10

**Published:** 2013-01-24

**Authors:** Isabelle Gaboury, Hélène Corriveau, Gilles Boire, François Cabana, Marie-Claude Beaulieu, Pierre Dagenais, Suzanne Gosselin, Earl Bogoch, Marie Rochette, Johanne Filiatrault, Sophie Laforest, Sonia Jean, Alvine Fansi, Diane Theriault, Bernard Burnand

**Affiliations:** 1 Department of Family Medicine and Emergency Medicine, University of Sherbrooke, Sherbrooke, QC, Canada; 2 Centre de recherche clinique Étienne-Le Bel, Sherbrooke, QC, Canada; 3 School of Rehabilitation, University of Sherbrooke, Sherbrooke, QC, Canada; 4 Centre de recherche sur le vieillissement, Sherbrooke, QC, Canada; 5 Department of Medicine, University of Sherbrooke, Sherbrooke, QC, Canada; 6 Institut national d’excellence en santé et en services sociaux, Montreal, QC, Canada; 7 Health and Social Services Center- University Institute of Geriatric of Sherbrooke, Sherbrooke, QC, Canada; 8 Keenan Research Centre, Toronto, ON, Canada; 9 St Michael’s Hospital, Department of Surgery, University of Toronto, Toronto, ON, Canada; 10 Ministère de la Santé et des Services sociaux du Québec, Québec, QC, Canada; 11 School of Rehabilitation, University of Montreal, Montreal, QC, Canada; 12 Centre de recherche de l’Institut universitaire de gériatrie de Montreal, Montreal, QC, Canada; 13 Centre for Research and Expertise in Social Gerontology (CRESG), CSSS Cavendish-Centre affilié universitaire, Montreal, QC, Canada; 14 Institut de recherche en santé publique de l’Université de Montréal (IRSPUM), Montreal, QC, Canada; 15 Institut national de santé publique du Quebec, Québec, QC, Canada; 16 Department of Health Community Sciences, University of Sherbrooke, Longueuil, QC, Canada; 17 Osteoporosis Canada, Toronto, ON, Canada; 18 University of Lausanne, Lausanne, Switzerland

**Keywords:** Fragility fracture, Osteoporosis, Fall prevention, Integrated program, Interorganizational collaboration, Canada, Controlled trial, Evaluation

## Abstract

**Background:**

Fractures associated with bone fragility in older adults signal the potential for secondary fracture. Fragility fractures often precipitate further decline in health and loss of mobility, with high associated costs for patients, families, society and the healthcare system. Promptly initiating a coordinated, comprehensive pharmacological bone health and falls prevention program post-fracture may improve osteoporosis treatment compliance; and reduce rates of falls and secondary fractures, and associated morbidity, mortality and costs.

**Methods/design:**

This pragmatic, controlled trial at 11 hospital sites in eight regions in Quebec, Canada, will recruit community-dwelling patients over age 50 who have sustained a fragility fracture to an intervention coordinated program or to standard care, according to the site. Site study coordinators will identify and recruit 1,596 participants for each study arm. Coordinators at intervention sites will facilitate continuity of care for bone health, and arrange fall prevention programs including physical exercise. The intervention teams include medical bone specialists, primary care physicians, pharmacists, nurses, rehabilitation clinicians, and community program organizers.

The primary outcome of this study is the incidence of secondary fragility fractures within an 18-month follow-up period. Secondary outcomes include initiation and compliance with bone health medication; time to first fall and number of clinically significant falls; fall-related hospitalization and mortality; physical activity; quality of life; fragility fracture-related costs; admission to a long term care facility; participants’ perceptions of care integration, expectations and satisfaction with the program; and participants’ compliance with the fall prevention program. Finally, professionals at intervention sites will participate in focus groups to identify barriers and facilitating factors for the integrated fragility fracture prevention program.

This integrated program will facilitate knowledge translation and dissemination via the following: involvement of various collaborators during the development and set-up of the integrated program; distribution of pamphlets about osteoporosis and fall prevention strategies to primary care physicians in the intervention group and patients in the control group; participation in evaluation activities; and eventual dissemination of study results.

**Study/trial registration:**

Clinical Trial.Gov NCT01745068

**Study ID number:**

CIHR grant # 267395

## Background

Among Canadians over 50 years of age, close to two in five women and one in six men will experience at least one fracture due to bone fragility (FF) [1-3], in other words a fracture resulting from minimal trauma or a fall that would not harm a healthy person. In women over age 50, the annual FF incidence rate is 14/1000, twice the incidence rate of cerebrovascular incidents and myocardial infarcts combined [4]. FFs cost Canada’s healthcare services over $2 billion annually [5,6], with costs expected to triple over the next 20 years [7,8].

Recent statistics reveal that about two million Canadians suffered from osteoporosis defined by bone mineral density (BMD) [9], but BMD is an imperfect measure of bone strength. Although FFs are generally related to bone fragility related to osteoporosis [1,10-13], and the relative risk for FF increases with decreasing BMD, the majority of FFs occur in individuals without BMD-defined osteoporosis. The occurrence of a FF is a sentinel event predicting a future FF [14], at least doubling the risk of secondary fractures compared with age- and BMD-matched individuals without a FF [15,16]. Indeed, men and women with a hip or spine FF are at high risk for repeat fractures, irrespective of their BMD results, which may be normal. Of note, about half of hip fracture patients have sustained previous FFs [17,18], suggesting that FFs are events that should guide preventive interventions. Hip fractures often precipitate decline in health or even death, and loss of mobility, with high associated costs for patients, families, society and the healthcare system [19-23].

The two mainstays of FF prevention are pharmaceutical interventions to strengthen bones, and fall prevention programs to maintain and improve strength and balance, including other strategies to reduce fall risk factors, keeping in mind that exercise also strengthens bones. The success of interventions to manage FFs more effectively, to identify and treat those with bone fragility, to maximize population health by preventing or delaying frailty, and to minimize costs [24-28] is mediated by the context of interventions within the local healthcare system [29,30]. Despite strong evidence of their effectiveness, prescription and adherence to osteoporosis treatment and fall prevention interventions following FFs remain sporadic and inconsistent in primary care. It is hoped that when post-fracture screening and fall prevention programs are systematically integrated, and secondary FFs may be prevented in an additive or even synergistic manner [30,31].

In the context of current primary care reform, secondary fracture prevention program research must evaluate multidisciplinary and interorganizational strategies, with attention to the regional context of implementation, the providers, and the participants. The present paper includes the design and evaluation procedures for a novel secondary FF prevention program, involving collaboration between secondary healthcare practitioners (orthopedic surgeons and medical bone specialists), primary care physicians (PCPs), group practice nurses, and local and provincial fall prevention programs, being implemented in the province of Quebec, Canada. This work builds on the Osteoporosis and Peripheral fractures: Treatment and Investigation through Multidisciplinary care at the CHUS (OPTIMUS) intervention. The proposed intervention involves PCPs in the evaluation and treatment of osteoporosis following FFs treated by orthopedic surgeons [32-38] and integrates local and provincial fall prevention programs implemented in the province of Quebec, including the Personalized Multifactorial Intervention (PMI), an individualized program offered to the most frail elders considered at risk for falls [39]; and a community-based fall prevention program aimed primarily at improving balance and strength through exercise for elders who have fallen or are concerned with falls (*Stand Up!*) [40]. This integrated program will be orchestrated by site study coordinators.

The overarching aim is to investigate the effectiveness, cost-effectiveness and cost-utility of an integrated FF prevention program, and to obtain a portrait of the barriers and facilitating factors for such programs. More specifically, the objectives are:

1. to combine existing fall prevention and post-fracture management programs in the province of Quebec into integrated interdisciplinary FF prevention programs;

2. to compare the performance of these integrated programs to results from control sites, using a pragmatic study design;

3. to identify barriers as well as factors that improve effectiveness across different implementation regions;

4. and to develop and engage in active knowledge transfer activities in Quebec regions where integrated FF prevention programs are neither adequately nor successfully implemented.

### Research questions and hypotheses

#### Primary question

Can an integrated FF program reduce the risk of a secondary FF in a population of patients 50 years of age and over, who sustained a FF 18 to 20 months previously?

#### Secondary questions

1) Can a post-FF management program be successfully integrated with PCPs’ health care, and local and national fall prevention programs, from a patient and healthcare system perspective?

2) Is this integrated FF prevention program effective and cost-effective?

3) What are the barriers and factors that improve effectiveness across different implementation regions?

Drawing upon the literature on integrated healthcare, fall and fracture prevention, as well as the OPTIMUS study [32-38], we hypothesize that an integrated FF program can reduce the risk of a secondary fracture by at least 30% in the population of interest. In addition, by examining the implementation process of the program, we will make recommendations to circumvent the barriers to implementation, and to build upon the factors that lead to improved integration of the care providers involved in the program.

#### Conceptual framework

The research program will draw on the key principles of the Development Model for Integrated Care (DMIC) [41-43] to implement the proposed intervention, and the Model of Structuration of Interprofessional Collaboration (MSIC) to explore interorganizational collaboration and its links to the selected clinical outcomes [44].

The DMIC is a recent model for the development of an integrated program [41-43] that has been validated through a panel of Dutch experts and is currently implemented within three settings in The Netherlands. This project will tap into the first and second phases of the DMIC model: Initiative and design; and Experiment and execution. These serve as a checklist for the development of the program in each intervention region, and as a guideline during the focus group discussions with the participating healthcare professionals involved in the intervention.

The MSIC lists 10 indicators, grouped into four dimensions: Shared goals and vision; Internalization; Formalization; and Governance. These indicators capture facets of relationships between individuals engaged in a collaborative practice, and the organizational context in which they work [44]. The MSIC has been validated in various healthcare contexts, mostly in the province of Quebec [45-47]. It will be used to guide the evaluation of collaboration among practitioners and organizations involved in the program (hospitals, orthopedists and medical bone specialists, PCPs in private practices, family medicine group (GMFs), and provincial and local fall prevention services).

## Methods

### Study design

A pragmatic, controlled trial design is being used to assess whether the intervention works in a setting that is as similar to routine practice as possible [48]. Within such a design, all participants are enrolled unless the study intervention might compromise patient safety; treating physicians and healthcare practitioners decide how the intervention is delivered; integrated models of healthcare are encouraged, as the intervention is delivered through the participation of a spectrum of practitioners in a range of healthcare organizations; and an intent-to-treat analysis is the only analysis possible [48-52].

### Setting

The study is taking place in eleven hospitals and various fall prevention programs, located in eight different administrative regions of the province of Quebec. Of these, six hospitals are offering the integrated program in four different regions (Sherbrooke, Lanaudière, and Montréal), while participants recruited from the other five hospitals (in the Outaouais, Trois-Rivières, Longueuil, Côte-Nord, and Amos regions) will constitute the standard care control group. The choice of these hospitals and regions is based on the variety of fall prevention programs available in the geographic area surrounding the selected hospitals [53]. The broad variety of sites will maximize the external generalizability of the study results, and is intended to optimize the potential identification of barriers to and facilitating factors for implementation of an integrated FF prevention program.

### Ethical approval

Given the large number of organizations involved, ethical aspects of this study were evaluated via a multi-center mechanism as recommended by the Ministère de la Santé et des Services Sociaux (MSSS). The Comité d’éthique de la recherche en santé chez l’humain du Centre hospitalier universitaire de Sherbrooke (CHUS) served as the principal ethics board and approved the final documents.

### Participants

The study population consists of community-dwelling males and females, who are 50 years of age and over, who have a PCP (this is the case for 94% of this age group in Canada [54]), who are able to follow simple instructions, and who have sustained a FF within two months of the recruitment date. Participants presenting with the following conditions will be excluded: fracture at sites not commonly associated with osteoporosis such as toe, finger, hand, foot, patella, head, and cervical spine; severe kidney insufficiency (grade 4 or 5); or an advanced stage of cancer or any other disease from which the patient is likely to die within the next year.

### Recruitment

Drawing upon the methodology of previous studies in a similar population [32-34,55], participants for intervention sites will be recruited by a study coordinator over a period of 12 months, and will provide their informed consent to participate and complete the baseline questionnaire. Three methods will be used to recruit intervention group participants:

1. Patients with FFs in participating outpatient orthopedic clinics will be identified by a study coordinator;

2. Hip fracture inpatients will be approached on the ward during their hospitalization;

3. And hospital emergency and ward discharge administrative data will be scanned for patients with a primary discharge code typical of a FF. These participants will be contacted by mail by the hospital to inform them about the research project and that a local study coordinator will contact them. Electronic discharge data allows patient identification within one week following discharge.

Due to budgetary and ethical considerations, only strategy 3 will be used to recruit participants in the control hospitals/regions. Should it be required as an alternative, FF patients’ identification through the *Régie de l’assurance maladie du Québec* (RAMQ) database will be considered, as in the Recognizing Osteoporosis and its Consequences in Quebec (ROCQ) study [55]. The main limitations of this alternative strategy as well as strategy 3 are a delay required to obtain patients’ information (estimated at two months in the case of the RAMQ data, one week for the hospital administrative data), and a potential selection bias (those more at risk for fractures, or who are younger and more educated may be more likely to consent); however, such strategies might be an efficient way to increase the external validity of the study by recruiting a broader range of fracture patients.

### Study interventions

#### Intervention group

Nurses working in the Family Medicine Groups and Cliniques-Réseau (in Montreal) (GMF/CRs) located in the areas served by the recruiting hospitals will receive a workshop on evidence-based pharmacological interventions for osteoporosis, and benefits of exercise and fall prevention strategies to prevent FF. All PCPs within the intervention group regions will receive a pamphlet summarizing the workshop.

Upon recruitment, FF participants will be educated by the study coordinator about osteoporosis as the cause of their FF, the importance of a rapid screening evaluation for potentially treatable primary causes of bone fragility, the benefits of early and prolonged treatment, the options for treatment to be adapted for individuals, and will be asked permission to contact their PCP and community pharmacist. Participants’ PCPs will receive written information containing a presumed osteoporosis diagnosis, investigations to be performed, correct interpretation of bone densitometry results in the context of a FF, treatment options adapted to the individual patient, and alternatives if the first prescriptions are not tolerated or are stopped.

The integrated intervention *per se* includes the following:

1. When possible, screening basic blood tests (*e.g*., calcium, phosphate, white blood cells, hemoglobin, platelets, vitamin D) in the context of a FF will be performed at the recruiting hospital laboratory, and the results will be transmitted to the participant’s PCP with a personal letter explaining the importance of seeing the patient rapidly (ideally within one month), and the urgency of initiating an osteoporosis treatment. If the PCP is part of a GMF/CR [56], then the clinic nurse will receive a copy of the information sent to the PCP.

2. Following a region-specific *a priori* defined algorithm to identify the most relevant resource for the participant, the study coordinator or the GMF/CR nurse (when possible) will orient the participant to an appropriate fall prevention program, and organize his/her registration or placement on the waiting list. A local exercise and fall prevention program appropriate for the patient according to the participant’s age, fracture risk, and abilities will be agreed upon by the study coordinator and the participant. The study coordinator will work with numerous local fall prevention champions supporting this research program, to establish and maintain an up-to-date list of available exercise and fall prevention programs at each site. The study coordinator will also encourage the participant to engage in additional physical activities at a comfortable level of intensity.

3. The participant will be called quarterly by the study coordinator or the GMF/CR nurse to monitor pharmacological treatment adherence, to detect and correct (if appropriate) inadequate intake or side effects affecting compliance, to answer questions, to encourage compliance, and to track fall and fracture information recorded in the participant diary.

4. The study coordinator or the GMF/CR nurse will communicate with the participant’s pharmacist at 6 and 12 months to confirm drug delivery to the participant (if the participant has been prescribed osteoporosis pharmacological treatment), and if possible to enlist this professional’s help in encouraging treatment.

5. At each follow-up, if the patient is not taking an adequate pharmacological treatment, a letter will be sent again to the PCP (and the GMF/CR nurse, if appropriate) suggesting that they treat the patient according to the participant’s 10-year fracture risk.

6. Participant adherence to the fall prevention program will be monitored at the mid-point and at the end of the program. If necessary, the study coordinator will arrange a program change to better fit the participant’s needs.

### Control group

The study coordinator will inform the participants about osteoporosis as the potential cause of their fracture. A lay pamphlet on strategies to prevent a secondary FF will be provided, including the recommendation to visit their PCPs and the need for fall prevention and pharmacological intervention in most patients.

### Outcomes and data collection

All participants will be followed for a period of 18 months. A multilevel approach is being used, in which outcomes at the patient level will be measured with quantitative tools, and outcomes at the organization level (*e.g*., perception of the degree of integration and collaboration between organizations) will be measured simultaneously using qualitative techniques [57]. Combining both qualitative and quantitative measures will allow a better understanding and contextualisation of the quantitative results, and verification as to how organization level factors impact the effectiveness outcomes [57,58].

### Quantitative data

With the exception of the first encounter with the participants recruited from the orthopedic clinics and the inpatients from the intervention sites with hip and other fractures, all data will be collected through phone surveys and the participant diary. A baseline questionnaire will be completed by all participants, including demographic data and relevant medical history (*e.g*., height, weight, smoking and drinking history, family history of hip fracture, use of glucocorticoids) and the CaMos questionnaire [59]. The Fracture Risk Assessment Tool (FRAX) score will be used, irrespective of BMD results availability [60]. A Canadian Association of Radiologists and Osteoporosis Canada 10-year absolute fracture risk (CAROC) score will be generated for participants who undergo a BMD test within +/− 3 months of the sentinel event [59,61]. A study exit questionnaire will be administered at the end of the intervention or when the participant withdraws his/her consent to participate.

### Primary outcome

The primary outcome is the incidence of secondary FF during the 18-month follow-up period, including the date of the event, related emergency room visit, and hospitalization. This will be ascertained during quarterly telephone surveys, and confirmed with the participant’s hospital record when possible.

### Secondary outcomes

Secondary outcomes will be assessed at 3, 6, 9, 12 and 18 months unless otherwise mentioned.

1. Initiation of osteoporosis treatment by the PCP (bisphosphonates or other effective osteoporosis drugs) [31], and compliance with osteoporosis treatment will be captured during telephone surveys. The data will be validated with the participant’s pharmacist via the medication possession ratio, which is measured from the first to the last prescription, with the denominator being the duration from index to the exhaustion of the last prescription and the numerator being the days supplied over that period from first to last prescription and the numerator being the total number of days in the interval [62].

2. Time to first fall, and number of clinically significant falls will be reported by participants from their diary of fall events, during telephone follow-up. This prospective method has been found to be reliable, minimizing recall bias, in other fall prevention studies [63].

3. Fall-related hospitalizations will be recorded in the participant diary and at each telephone follow-up, and validated with the participant’s hospital records.

4. FF-related death will be confirmed from the RAMQ administrative database.

5. Participants’ quality of life will be documented bi-annually during telephone surveys, using the EQ-5D-3 L questionnaire [64]. This is an extensively validated questionnaire that provides a global measure of the respondent’s health status and quality of life [65-67].

6. Practice of physical activities will be measured using the Community Healthy Activities Model Program for Seniors (CHAMPS) [68,69]. This tool has demonstrated very good psychometric properties and sensitivity, even with low intensity physical activities.

7. FF-related costs will be captured over the study duration. Medical costs from the participant’s hospital record include: medical assessment, prescription and non-prescription drugs; and resource use related to the management of the FF and/or long-term complications, including hospitalization. Also, lost days of work, annual income (if the participant is in the labor force), travel to the hospital and/or rehabilitation facilities, parking, and out-of-pocket expenses for drugs and rehabilitation devices (*e.g*., splint) will be compiled. Protocol-driven costs will be excluded, with the exception of the costs related to the participant’s fall prevention program.

8. Admission to a long-term care facility will be queried during each telephone survey.

9. All participants’ perceptions of care integration will be captured in the exit questionnaire. In the absence of a formally validated instrument to measure the outcome of interest [70], the measurement tool will consist of a customized version of a questionnaire on the perception of care integration from a patient perspective [71]. Five questions, corresponding to the interaction subscale, are being used to investigate this study objective on program integration.

10. Intervention participant’s satisfaction with the FF prevention program will be measured using questions similar to those used by Berendsen *et al*. for their assessment of the sources and amount of information preferred by the participant in the context of an integrated program [72], along with general questions with regards to the participant’s satisfaction with the program.

11. Participants’ expectations and whether they are perceived as met will be collected at the beginning and end of the program. The questionnaire is an adapted version of Gignac’s Program Expectation Scale and uses a 3-point scale ranging from 1 (not at all likely) to 3 (extremely likely) [73]. Participants will also be asked to rate the extent to which they believe the program would produce (before the program) or has produced (end of the program) health benefits for themselves.

12. Individuals who chose to leave the program (or part of it) will be contacted by telephone to document the reason(s) for withdrawing while completing the exit questionnaire.

### Qualitative data

Focus group sessions organized per intervention region will gather a purposeful sample of healthcare managers of fall prevention programs, medical bone specialists, orthopedic surgeons, PCPs and GMF nurses involved in the trial, as well as patients. The sessions are scheduled to last about 60 minutes in length. A maximum of eight professionals will participate per focus group, so sessions with healthcare professionals will be repeated twice for greater participation. The topics and questions of the semi-structured questionnaire will be guided by the conceptual frameworks selected for this project [41,44]. The first session (at baseline) will aim to understand both the factors for success and the potential barriers to the implementation of a FF prevention program. The second session will target the barriers and facilitating factors for maintenance of a FF prevention program, as well as measure the degree of integration between the organizations involved (at 12 months). The two focus groups for the patients (one at baseline and another one between 12 and 18 months post-recruitment) will be geared towards patients’ expectations and satisfaction about the program, as well as perception of integration of care. All focus group sessions will be audiotaped and transcribed.

### Statistical analysis

#### Sample size

##### Quantitative evaluation of entire cohort

Based on preliminary (almost four years follow-up) results from the OPTIMUS project, the incidence of a secondary fracture at 16 months in the control group is expected to be 10%; therefore, this incidence of fracture was used as a proxy for the sample size calculation of this project (primary outcome measured at 18 months). Considering the reported efficacy of antiresorptive drugs among others (average absolute reduction rate of 4%) [74-77], the results obtained in the intervention group of the OPTIMUS cohort (expected absolute reduction rate in FF of 2.5%), as well as the expected synergy with fall prevention programs, and collective orders that will allow for a rapid pharmacological intervention, an absolute reduction of 3% (*i.e*., a secondary fracture incidence at 18 months of 7% in the intervention group) is a conservative estimate of the effect to be detected. Under these assumptions and assuming a power of 80% and an alpha level of 5%, 1,356 participants per group are necessary to verify the primary hypothesis. It is estimated that approximately 15% of the participants will be lost to follow-up or will withdraw from the study prior to the 18-month data collection time point. Therefore, a total of 1,596 participants will be recruited in each group (total N = 3,192).

##### Qualitative focus group analyses

Based on the qualitative literature, it is estimated that 6 to 12 participants are required to achieve study themes redundancy [78,79]. Our targeted sample size should then allow for the saturation of the various barriers to and facilitators of the implementation of an integrated FF prevention program (first sessions’ focus), as well as the qualitative assessment of the intensity of interorganizational collaboration within each intervention site (second sessions’ focus) [80,81].

### Data analysis

#### Quantitative component

Descriptive summaries of baseline participant characteristics in the control and intervention groups will be generated. Any statistical differences (due to selection bias, for example) between the study groups or within a study group (between the different sites) will be tested using chi-square tests or Student’s t-tests (or their non-parametric equivalents if necessary) and accounted for in the following analyses. According to the pragmatic design standards, statistical analysis of the data will follow the intention-to-treat principle; *i.e*., the analysis will include all patients regardless of their compliance with their study group intervention. The implementation process will also be documented with descriptive statistics.

#### Primary outcome

The numbers of participants presenting with a least one secondary FF at 18 months will be compared using a chi-square test. A logistic regression model will be fit to assess the intervention effect, while adjusting for variables thought to influence the outcome and accommodating any imbalance between groups at baseline. The intervention effect and its 95% confidence interval will be generated. Descriptive statistics on the cumulative incidence of recurrent FF will also be generated for each group.

#### Secondary outcomes

Initiation and compliance with pharmacological osteoporosis treatment at 3, 6, 9, 12 and 18 months, and mortality rate will be analysed independently, as for the primary outcome. Time to first fall will be compared between study groups using a time-to-event type of analysis (log-rank test or Gehan-Wilcoxon, depending on the data distribution). A Cox regression model that allows for the adjustment of baseline variables that have been shown to influence the outcome will be used. The difference in the number of significant falls and fall-related hospitalizations will be assessed using a Poisson regression model. Participants’ quality of life and practice of physical activities measured over time will be fit and compared between the two study groups using an analysis of variance (ANOVA) for repeated measures. The total score of the participant’s perception of care integration will be generated for each participant by summing up all questionnaire items. Group scores will be compared using a linear regression model. If numbers allow, a sub-group analysis will be considered among the intervention group to test the effect of provincial versus local fall prevention programs as a covariate on the primary outcome.

The cost-effectiveness ratio will be expressed as the difference in costs between the study groups, divided by the difference in secondary fracture rates between the two groups. Costs will be calculated as the units of resources used in the treatment of a FF, multiplied by the cost of one unit. The uncertainty concerning the incremental cost, the incremental effectiveness, and the incremental cost-effectiveness ratio will be estimated by conducting probabilistic analysis through non-parametric bootstrapping [82]. Results from the bootstrapping exercise will be depicted by cost-effectiveness acceptability curves. All quantitative analyses will be performed with the SPSS 18 software (SPSS, Inc., 2009, Chicago, IL), with the exception of the economic analyses, which will be performed using Oracle Crystal Ball (http://www.oracle.com/us/products/applications/crystalball/index.html).

#### Qualitative component

Focus group audio tapes and transcripts will be reviewed simultaneously to assess validity of the transcription process. An interpretive approach will inform the analysis of the transcript [83,84]. Two examiners will read the transcripts independently, and a thematic analysis will be conducted to identify themes that emerge, that correspond to the study questions. Codes will be compared and discussed until an acceptable inter-judge reliability is obtained [83]. Data analysis will be conducted using the scoring scheme proposed by D’Amour and colleagues [44,85], and supported by the NVivo software. An audit trail will be compiled to record the steps taken and decisions made during the analytical process, which will ensure the reproducibility of the analysis. Data saturation will be considered to have been reached when a theme has been discussed by at least six interviewees [78]. Analysis will examine coherence and differences among the focus group participants, and will be discussed within a small subgroup of the research team. Integration with the quantitative data will be considered, especially for explanation of any foreseen variation in the primary outcome between the intervention sites.

#### Knowledge translation and dissemination

Several activities will serve to meet the fourth objective of this project related to knowledge translation throughout the intervention [86]:

1. A panel of volunteer healthcare professionals involved in the treatment of FF and public health, as well as a patient representative from the intervention regions, will design the pamphlets for the control participants, and for the intervention PCPs. Webminars will also be offered to healthcare professionals in the intervention regions. This will be done through a modified Delphi survey [87-89] with refinement using a focus group including a maximum of 12 participants. This initiative will create expectations among the professionals and their communities, while sensitizing them to the clinical and economic burden of FFs. This strategy will also empower these professionals vis-à-vis the research team, thus creating a fertile environment for active collaboration and knowledge transfer during the trial.

2. The personalized letter sent to the intervention group participants’ PCPs is an excellent opportunity for knowledge transfer and continued education. When carefully designed, this mode of communication between secondary and primary care has shown benefits, especially with the older adult population [90-92].

3. A final report as well as an information session will be prepared for the MSSS and the Institut national de santé publique du Québec (INSPQ) decision makers and managers to inform them about the study results and to promote the sustainability and expanded geographical coverage of the program.

4. We anticipate that the process of evaluation of the currently available fall prevention programs will affect the willingness of managers to pursue evaluation of the organization (*e.g*., hospitals, medical group practices, provincial and local fall prevention programs) and delivery processes of their programs. Such a bottom-up approach might eventually improve the knowledge translation and dissemination of the proposed study recommendations with the confidence that these will have a longer and measurable impact upon the organizations involved. Moreover, the involvement of collaborators committed throughout major networks of action among the government and osteoporosis knowledge transfer groups should facilitate the translation of the results among the population and stimulate the creation of other similar initiatives in the province.

5. Conventional knowledge translation strategies such as presentations to national and international conferences and publications in peer-reviewed open-access journals will also be undertaken.

## Discussion

This trial is testing a comprehensive, integrated secondary FF prevention program in a real-life setting, and is designed to optimize translation of research evidence into practice [93-95]. The pragmatic evaluative design of this project, rooted in the Canadian Institutes of Health Research Integrated Knowledge Translation process [96], will provide estimates of the effectiveness of a FF prevention program in different socioeconomic contexts, thereby increasing the generalizability of the results.

FF prevention is needed for much longer time periods than the length of this study, and the creation of a cohort of patients who have experienced FFs offers an opportunity to further address continuity and gaps regarding integrated FF prevention programs. The study team is seeking funding for continuing research, including follow-up at five years.

A limitation of this study lies in the recruitment strategies. Patients in the control group, identified through administrative databases, may present with more severe risk factors, which translate into a higher likelihood of a secondary FF. This may create a selection bias between the recruiting sites, which will be accounted for in the statistical analyses. In the intervention group, a surveillance bias for secondary FF is possible, since this group is sensitized to FFs. This may reduce the observed study effect; however, the large size of this trial, based on conservative effect estimates, should provide sufficient power to detect the intervention effect, if present. Finally, control sites/regions will be monitored throughout the trial to identify emerging fall prevention programs. This will be accounted for in the statistical analysis should participants take part in these programs. Another limitation related to the external validity of this study lies in the organizational variations between participating regions. However, the qualitative component of the study should shed light on those variations and help in a more accurate interpretation of the results.

The number of aging baby boomers, increasing longevity, and higher standards for quality of care are combining to create the ‘perfect storm’ of healthcare issues that will emerge over the coming decades. Rapidly increasing numbers of FFs are a significant concern for older adults, and place enormous demands on already stretched health resources. Solutions to slow this ‘epidemic’ have already been developed and tested in practice: falls can be prevented through existing programs; cost-effective drugs decrease the risk for fractures in high-risk groups; and care following FFs is improving. Nevertheless, the impact of these interventions is blunted by low patient participation and poor intervention integration.

This novel integrated FF prevention program aims to improve interorganizational collaboration and communication between both primary and secondary healthcare practitioners. As we help health professionals to improve the continuum of care and patient-centered care, the goal is also to improve chronic disease prevention and management.

## Abbreviations

BMD: Bone mineral density; CHUS: Centre hospitalier universitaire de Sherbrooke; CR: Clinique-réseau; DMIC: Development Model for Integrated Care; FF: Fragility fracture; GMF: Family medicine group; INSPQ: Institut national de santé publique du Québec; MSIC: Model of structuration of interprofessional collaboration; MSSS: Ministère de la santé et des services sociaux; OPTIMUS: Treatment and investigation through multidisciplinary care at the CHUS; PCP: Primary care physician; PMI: Personalized multifactorial intervention; RAMQ: Régie de l’assurance maladie du Québec; ROCQ: Recognize osteoporosis and its consequences in Quebec.

## Competing interests

The authors declare that they have no competing interests.

## Authors’ contributions

All authors participated in the conception and design of the study. IG drafted the manuscript. All authors read and approved the final manuscript. The funding body has not been involved in design, in the collection, analysis, and interpretation of data; in the writing of the manuscript; and in the decision to submit the manuscript for publication. All authors read and approved the final manuscript.
